# Porosity Engineering of Dried Smart Poly(*N*-isopropylacrylamide) Hydrogels for Gas Sensing

**DOI:** 10.1021/acs.biomac.3c00738

**Published:** 2023-12-04

**Authors:** Sitao Wang, Chen Jiao, Gerald Gerlach, Julia Körner

**Affiliations:** † Institute of Solid-State Electronics, Dresden University of Technology, 01062 Dresden, Germany; ‡ Leibniz-Institut für Polymerforschung Dresden e.V., Hohe Straße 6, 01069 Dresden, Germany; § Institute of Electrical Engineering and Measurement Technology, Leibniz Universität Hannover, 30167 Hannover, Germany

## Abstract

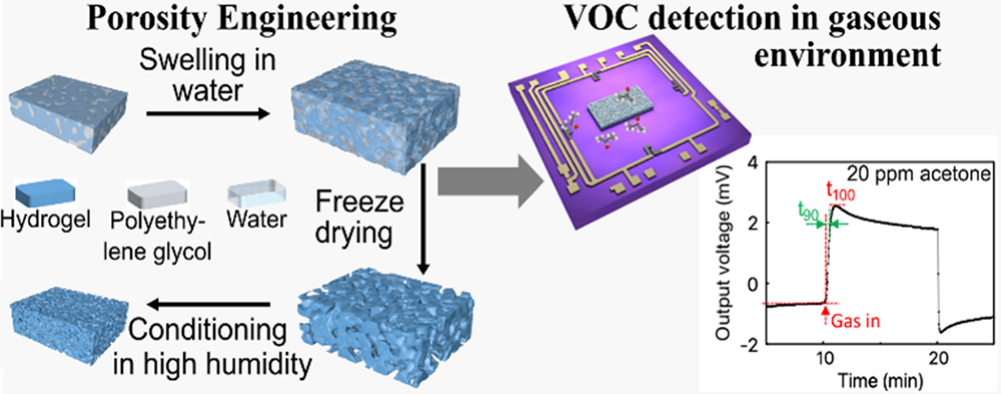

A recent study unveiled the potential of acrylamide-based stimulus-responsive hydrogels for volatile organic compound detection in gaseous environments. However, for gas sensing, a large surface area, that is, a highly porous material, offering many adsorption sites is crucial. The large humidity variation in the gaseous environment constitutes a significant challenge for preserving an initially porous structure, as the pores tend to be unstable and irreversibly collapse. Therefore, the present investigation focuses on enhancing the porosity of smart PNiPAAm hydrogels under the conditions of a gaseous environment and the preservation of the structural integrity for long-term use. We have studied the influence of polyethylene glycol (PEG) as a porogen and the application of different drying methods and posttreatment. The investigations lead to the conclusion that only the combination of PEG addition, freeze-drying, and subsequent conditioning in high relative humidity enables a long-term stable formation of a porous surface and inner structure of the material. The significantly enhanced swelling response in a gaseous environment and in the test gas acetone is confirmed by gravimetric experiments of bulk samples and continuous measurements of thin films on piezoresistive pressure sensor chips. These measurements are furthermore complemented by an in-depth analysis of the morphology and microstructure. While the study was conducted for PNiPAAm, the insights and developed processes are general in nature and can be applied for porosity engineering of other smart hydrogel materials for VOC detection in gaseous environments.

## 1 Introduction

Volatile organic compounds (VOCs) in human exhaled breath offer a noninvasive approach to monitoring general health and biomarkers for diseases, such as cancer, diabetes, or respiratory issues. Specifically, they are easily accessible and offer high sensitivity and low response times compared to other techniques such as blood, urine, and feces analysis.^1−5^ However, tracking specific molecules of interest in exhaled breath is challenging due to the typically low molecular concentrations [at or below parts-per-million (ppm) and parts-per-billion levels (ppb)]. Moreover, the presence of many different and often similar molecules or compounds and the high probability of cross influences may obscure the actual amount.^4,5^ To date, the most accurate breath analysis approaches rely on sample collection in a bag, tube, or container or mask and subsequent analysis based on gas chromatography, mass spectrometry, or infrared spectroscopy.^6−9^ These methods are very precise down to ppb levels but also costly and require extensive equipment and trained personnel.^6^ Potentially miniaturizable concepts based on metal oxide semiconductor materials, field effect transistors, chemiluminescence, or laser photoacoustic spectroscopy could enable real-time analysis without sampling or pretreatment, making them also suitable for continuous self-monitoring.^6,10−13^ However, to date, these approaches often lack sensitivity, selectivity, and stability.^6^ Consequently, many efforts are targeting the development of sensors and sensor arrays for exhaled breath analysis, which combine the high sensitivity and selectivity of laboratory-based approaches with the ease of use and potential for miniaturization of smaller and wearable devices.^14−17^ In order to achieve that, a crucial part is the engineering of the sensing material required for the detection of the analyte of interest.

Stimulus-responsive, that is, smart, hydrogels are very interesting candidates for this purpose. In contrast to their nonresponsive counterparts, which are commonly used in agricultural,^18−20^ biomedical,^21−25^ and pharmaceutical contexts,^26−28^ smart polymers are capable of reversibly changing their volumetric swelling state. This is triggered by an external physical or chemical stimulus, such as temperature, light, electrical field, pH, ionic strength, or specific molecules.^29−43^ The responsiveness is due to the functional groups and moieties attached to the polymer backbone, which offers great potential for tailoring the hydrogels for high selectivity and specificity for target analytes in sensing and actuating purposes. As the swelling response is mainly mediated by the uptake and release of liquid, most developments of smart hydrogels have focused so far on liquid environments.

In order to extend the application space and harness the potential of smart hydrogels for VOC detection, we have investigated their ability to maintain a reversible and detectable volume change in gaseous environments. Therefore, our previous work focused on screening different synthetic acrylamide-based materials for their swelling response in air with varying relative humidity from 5 to 100% and the exemplary VOCs acetone and isopropanol.^44^ From the studied materials polyacrylamide (PAM), poly(*N*-isopropylacrylamide) (PNiPAAm), poly(acrylic acid) (PAA), and various copolymer combinations, PNiPAAm was identified as the most suitable candidate, which exhibited the highest and most reproducible response for acetone detection. However, the relatively dense polymer structure provides a limited surface area, resulting in a slow response to the target gaseous analyte. Moreover, due to the intrinsic polymer relaxation processes as a consequence of different environmental conditions, irreversible collapse of an originally porous surface and inner structures occurs frequently, adversely affecting the swelling response. Thus, tailored microstructural modifications aimed at increasing the surface area and number of adsorption sites are crucial for improving the sensing performance. This furthermore includes the stabilization and preservation of a created porous structure in the conditions of a gaseous environment.

A variety of methods to tailor porosity have been studied and developed for hydrogels in liquid environments, mainly for tissue engineering and drug delivery.^45,46^ Given the inherent porosity of hydrogels resulting from the molecular characteristics of their precursors and the fabrication conditions, several methods, such as particle leaching, phase separation, 3D printing, electrospinning, and cryotemplating, have been extensively explored and utilized to introduce additional voids within the material matrix. These approaches are commonly employed to create porous hydrogels across various scales.^47^ Comprehensive overviews by Foudazi et al. and Nicol highlight the features of each method, achievable pore sizes, and distributions as well as discuss the underlying theoretical background related to the different states of a hydrogel.^47,48^ However, almost all application cases relate to liquid environments and only very few studies employ porosity-engineered hydrogels in a somewhat nonliquid surrounding, for example, in solar water purification.^49^ To the best of our knowledge, nothing has been reported for porosity engineering of hydrogels for gas sensing applications.

It is therefore the aim of the present work to investigate how the porosity of smart hydrogels can be engineered to achieve an improved swelling response in gaseous environments.

There are three aspects associated with this:

1. Creation of pores: Similar to liquid applications, a porous structure increases the surface area and number of available binding sites for the gas molecules. In our study, we employ a porogen to the precursor solution, which acts as a molecular imprint during the polymerization process and is removed afterward.
2. Internal and surface porosity: While a porous structure inside the hydrogel is fairly stable, the pores on the surface tend to close due to reduced mechanical support from the interface with the surrounding medium. For gas sensing, it is crucial to preserve an equal porosity on the surface and within the bulk part of the material to maintain a large surface area for adsorption and enable a fast response.
3. Stabilization of the porous structure: In contrast to the common use cases where the hydrogel is immersed in liquid, the gas atmosphere with varying relative humidity over the full spectrum from very low (∼1%) to saturated (100%) poses a challenge for the preservation of structural integrity of the material. Due to the drying processes and associated mechanical forces in the gas atmosphere, porous structures can irreversibly collapse.^50^

In the following, we present a study on how to create a tailored and stable porous inner and surface structure for enhancing the gas-sensing properties of smart poly(*N*-isopropylacrylamide) (PNiPAAm) hydrogels. The addition of polyethylene glycol (PEG) as porogen and the use of different drying methods are investigated to address the aforementioned aspects and challenges.

Two different types of samples are used for this purpose: bulk and on-chip smart PNiPAAm hydrogels. With the bulk samples, we study the influence of PEG and various drying methods on the creation and stabilization of porosity and compare the appearance and microstructure of differently treated samples with optical and scanning electron microscopy. The swelling response to different relative humidity conditions and with the addition of 100 ppm of organic solvent acetone as test analyte gas is determined by weighing measurements which is limited to the study of equilibrium (either fully swollen or shrunken) states.

In order to also investigate the dynamic swelling responses, thin films of the same hydrogel materials as before are fabricated on piezoresistive pressure sensor chips and subjected to varying concentrations of acetone. Through the pressure sensor, time-dependent swelling curves can be obtained. Furthermore, since the samples are attached to a surface, the interplay between porosity and adhesion properties can be investigated.

All experiments have been performed with the exemplary test analyte gas acetone at room temperature (22 °C) as the focus of the study is to investigate approaches to achieve a stable porous surface and internal structure of a smart hydrogel material in a gaseous environment.

## 2 Experimental Section

The presented study focuses on the porosity engineering of PNiPAAm hydrogels to achieve (i) enhanced gas sorption rates, (ii) larger and faster volume changes, (iii) equally porous structures on the surface and in the bulk part, and (iv) long-term stability of the pores under changing conditions of liquid and gaseous environments with varying relative humidity. In the following, the relevant materials, experimental processes, and methods are described.

### 2.1 Materials

*N*-isopropylacrylamide (NIPAAm), *N,N’*-methylenebis(acrylamide) (MBA), ammonium persulfate (APS), *N,N,N′,N’*-tetramethylethylenediamine (TEMED), and lithium phenyl-2,4,6-trimethylbenzoylphosphinate (LAP) were purchased from Sigma-Aldrich (Germany) and were used as received. Polyethylene glycol (PEG) with different molecular weights was purchased from Fluka (Switzerland) and used as received.

### 2.2 Hydrogel Synthesis

**Figure 1:**
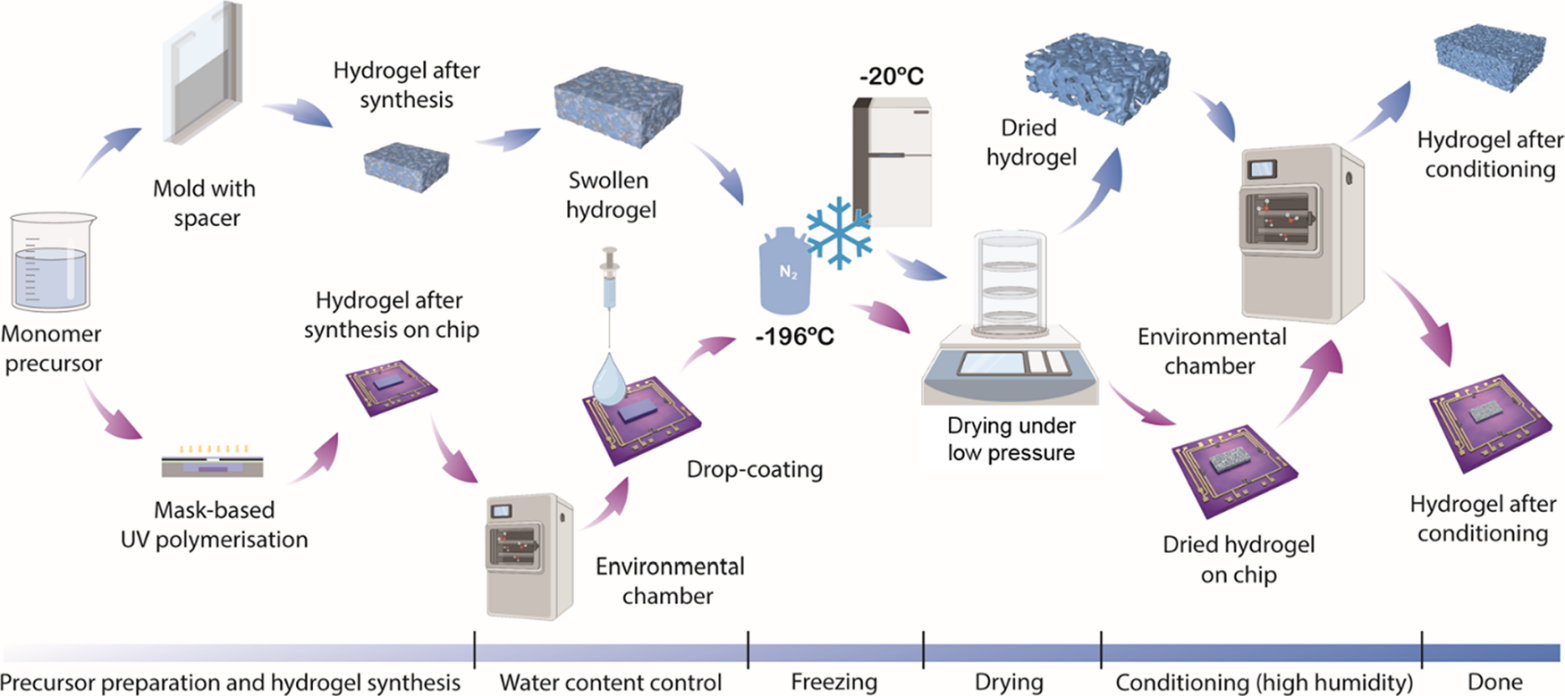
Fabrication process for bulk (blue) and thin-film hydrogel layer on the chip (purple). This figure was partially created by Figdraw.

Samples for the conducted studies were fabricated by the following steps: Preparation of precursor solution, polymerization either as bulk or patterned thin films on a pressure sensor chip, washing, drying, and subsequent conditioning, as shown in Figure 1. The details are outlined in the following.

#### 2.2.1 Unmodified Bulk PNiPAAm

Samples were prepared using free-radical polymerization initiated by the anionic initiator APS. First, NiPAAm (4.42 mmol, 0.5 g) and MBA (0.21 mmol, 0.033 g) were fully dissolved in 3 mL of deionized water and degassed with nitrogen for 5 min. By adding TEMED (0.05 mmol, 7.5 μL) and APS (0.07 mmol, 0.0167 g), the precursor solution was obtained. Hydrogel sheets (66 mm × 42 mm × 500 μm) were prepared by pipetting the precursor solution into a mold consisting of two microscope glass slides (76 mm × 52 mm) with a 500 μm thick Teflon spacer (Hightechflon GmbH & Co. KG, Germany) in between. Edges of the mold were secured by binder clips (19 mm) to keep liquid from escaping. Free radical polymerization was initiated in an ice bath by adding APS and was allowed to continue for 3 days in the refrigerator at 4 °C. The cooling is required to achieve a homogeneous polymer structure since the exothermal chemical reaction during polymerization can lead to local phase separations when the transition temperature of PNiPAAm is exceeded.^51^

The polymerized hydrogel sheets were removed from the mold and immersed in deionized water for another 3 days (with daily exchange of solution) in order to remove all unreacted components.

#### 2.2.2 PEG-Modified Bulk PNiPAAm

The fabrication of the PEG-modified PNiPAAm hydrogel followed the same procedure as described above for the unmodified samples but with two alterations: As an initial step, 1 g of PEG was dissolved in 3 mL of deionized water and then all other reactants were added. Following the identical degassing process, the resulting transparent mixture was injected into the same mold, but polymerization was achieved by simply storing the samples in cleanroom conditions (22 °C, 45% relative humidity) for 24 h without additional cooling.

Besides the PEG addition, the local phase separations due to the reaction temperature further increase the porosity of the polymerized material.^52,53^

After synthesis, the PEG-modified hydrogel sheets were soaked in deionized water for 30 days with a daily water exchange. The longer washing time (compared to 3 days for unmodified samples) was chosen since the presence of PEG impacts the conversion efficiency of NIPAAm monomers. It drops from 96.5 wt % for pure PNiPAAm to approximately 60 wt % for a 2:1 PEG to monomer ratio.^52^ Hence, more unreacted material may be present after polymerization, and therefore, longer washing was conducted to ensure complete removal thereof as well as the PEG.

All experiments, except the study on the influence of PEG molecular weight, have been carried out with PEG 10,000 g mol^–1^.

#### 2.2.3 Patterned PNiPAAm on Pressure Sensor Chip

In addition to bulk samples, patterned unmodified and PEG-modified PNiPAAm hydrogels were synthesized on a piezoresistive pressure sensor by mask-based UV photopolymerization for automated investigation of swelling properties and detection of small volume changes. For UV polymerization, NiPAAm (4.42 mmol, 0.5 g), MBA (0.21 mmol, 0.033 g), and 1 mol % LAP (0.0442 mmol, 0.013 g) were fully dissolved in deionized water and degassed with nitrogen for 5 min. The resulting precursor solution was transferred into an amber glass vial and stored in a refrigerator (at 4 °C) for further use. The PEG-modified precursor was obtained by dissolving 1 g of PEG in 3 mL of deionized water as the initial step, as described in Section 2.2.

To achieve the desired hydrogel geometry on the pressure sensor membrane, the sensor chip was immersed in a mold filled with precursor solution, and a thin hydrogel layer of predefined shape (rectangular) and thickness polymerized by 20 s of UV light exposure through a patterned photomask. The mold depth is 150 μm, resulting in a polymerized hydrogel thickness of (1–5) μm in the dried state. The mask has been made from AGFA CAMERA CE film (thickness of 100 μm) with a black coating to block any UV light through the unopened parts. In order to avoid overpolymerization and localized phase transition of the hydrogel due to heating induced by the UV lamp, the monomer concentration was reduced to the value given above (originally it was twice that amount). Additionally, the UV chamber was intermittently cooled by switching off the lamp and placing ice bags inside.

To remove all unreacted components as well as the PEG for the modified PNiPAAm, the chips were washed in deionized water for 3 days with daily solution exchange and then dried. Please note that the much thinner and smaller on-chip hydrogels require a much shorter rinsing time than for the bulk samples. After the completion of sample fabrication, the chip was mounted to a printed circuit board and electrically connected. Further fabrication details of hydrogels on pressure sensor chips are described in Figure S3 and in ref (54).

### 2.3 Drying of Synthesized Hydrogels

We employed various drying methods and protocols for both bulk and patterned hydrogels on chip to study the influence on the hydrogel porosity.

#### 2.3.1 Air-Drying

Samples were stored under ambient cleanroom conditions (22 °C, 45% relative humidity) for 3 days to ensure complete removal of any liquid. Please note that the drying process does not aim to completely eliminate all of the water molecules from the material. Instead, it continues until the material reaches equilibrium with the clean room conditions, ensuring comparability of samples.

#### 2.3.2 Freeze-Drying of Bulk Hydrogel Samples

To obtain a dried three-dimensional network structure from bulk watery hydrogels, the as-prepared samples were frozen at various cooling temperatures and then freeze-dried under reduced pressure to preserve the porous network. Therefore, hydrogels were put into 50 mL glass vials (fitting to the connectors of the freeze-dryer [Christ ALPHA 1–4 LD plus, Martin Christ, Germany)] after reaching swollen equilibrium in deionized water. One sample group was then immersed in liquid nitrogen (−196 °C) for 10 min and moved to the freeze-dryer. The other group was stored in a freezer at −20 °C (Liebherr, MediLine, Germany) for 24 h and then put into a freeze-dryer. All samples were connected to the freeze-drying equipment for 24 h to ensure complete sublimation of ice crystals (Figure 1). After being removed from the freeze-dryer, the samples were stored in the ambient air environment of the clean room to ensure the same initial equilibrium state as the air-dried ones.

#### 2.3.3 Freeze-Drying of Hydrogel on Chip Samples

In order to ensure stable adhesion of the patterned hydrogels on the pressure sensor chips after freeze-drying, these samples were stored in a sealed environmental chamber (HC 0020, Heraeus Industrietechnik, Germany) with saturated water vapor atmosphere for another 3 days after the 3-day rinsing. Right before the above-mentioned freezing procedure with different cooling rates in liquid nitrogen or in a freezer, the hydrogel samples were wetted by pipetting a water droplet on top (drop-coating). After 1 min, the residual water was removed by a cleanroom tissue, and the samples were freeze-dried with the same protocol as the bulk hydrogel.

### 2.4 Conditioning of Samples

The samples were freeze-dried in a swollen state. In order to reach a stable state for the subsequent experiments, all samples were therefore placed into an environmental chamber (HC 0020, Heraeus Industrietechnik, Germany) with >99% relative humidity for 3 days to allow the polymer chains to relax and reach an equilibrium. After that, samples were either directly used in experiments or stored under cleanroom conditions for further use.

### 2.5 Swelling Studies

#### 2.5.1 Gravimetric Measurement of Bulk Samples

The response of bulk samples to varying relative humidity (RH) from zero percent to saturated (RH = [0; 20; 40; 60; 80; 100; *100] %) with and without the addition of 100 ppm of acetone as test analyte gas is characterized by weighing measurements. Please note that we distinguish between 100% RH created by a humid gas flow and a saturated 100% condition created by a water reservoir in the environmental chamber. The latter is denoted as *100% RH for the remainder of the text and refers to an excess abundance of free water vapor molecules. In contrast to that, the amount of water molecules is still limited to 100% RH.

For the measurements, fabricated samples are broken into small pieces (thickness 500 μm; irregular shape as precise cutting is not possible; (5–10) mg per piece) after conditioning. They are exposed to an environmental condition for 24 h, weighed (Mettler Toledo XP26 analytical balance, sensitivity 1 μg), and then left to recover for another 24 h under ambient cleanroom conditions before the next measurement step.

A detailed description of the weighing procedure and creation of the gaseous environment can be found in ref (44) and the Supporting Information.

A hydrogel sample’s normalized relative weight change *W*_r_ in response to the environmental condition can be calculated by
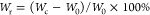
1where *W*_c_ and *W*_0_ denote the sample weight after exposure to a predetermined gas atmosphere and in the initial state, respectively. The delta relative weight change Δ*W*_r_ in response to additional solvent is obtained by subtracting the normalized relative weight change for solely relative humidity *W*_r,RH_ from the values for relative humidity plus solvent *W*_r,RH+VOC_, hence:
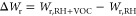
2Each sample was weighed three times, and the mean values and standard deviation were calculated accordingly.

#### 2.5.2 On-Chip Sample Response to Varying Acetone Concentration

The pressure sensor chips equipped with the hydrogel sample are placed in a sealed chamber with a nitrogen atmosphere, and the desired environmental condition is created by injection of liquid acetone.^55^ In the presented studies, the acetone concentration is varied from 20 to 100 ppm in steps of 20 ppm, corresponding to an injected volume of (0.02/0.04/0.06/0.08/0.10) mL.

The sample is subjected to each environmental condition for 10 min, followed by another 10 min of rinsing with dried nitrogen flow to allow the material to recover before the next measurement step.

Sample swelling exerts a force on the sensor membrane it is adhered to, leading to increased bending and, hence, resistance of the integrated piezoresistive pressure sensors.^56^ The resulting change in the chip’s output voltage was recorded with a digital multimeter (Fluke 45 Dual Display Multimeter, Germany).

For these experiments, temperature and pressure fluctuations in the cleanroom as well as gas condensation on the chamber walls are negligible.

### 2.6 Structural and Compositional Sample Analysis

Optical and scanning electron microscopy (SEM) were used to study the sample appearance and microstructure (Leica MZ6 stereo microscope and Zeiss Supra 40 VPF). For SEM analysis, samples were sputter-coated with 10 nm of gold (SC7620 mini Sputter Coater, Polaron, Germany) to avoid charging effects due to the polymer’s low electric conductivity. Please note that only fully dried samples can be investigated by SEM while optical microscopy images can also be taken in the wetted state.

Thickness measurements of on-chip samples were obtained by confocal microscopy and laser scanning (μSurf and μScan; NanoFocus AG, Oberhausen, Germany).

To study the PEG content within the hydrogel, samples were analyzed by FT-IR spectroscopy (Bruker Vertex 80v, Ettlingen, Germany) coupled to the attenuated total reflection (ATR) method. The IR spectra were recorded in the region of 4000–600 cm^–1^ with a total accumulation of 100 scans at a resolution of 4 cm^–1^. For spectra processing, including baseline correction and normalization, the machine’s *OPUS* software was employed.

## 3 Results and Discussion

The hydrogel samples were fabricated and characterized as described in Section 2. In the following, the corresponding results are presented and discussed.

### 3.1 Bulk Hydrogel Samples

#### 3.1.1 Appearance and Microstructure

**Figure 2:**
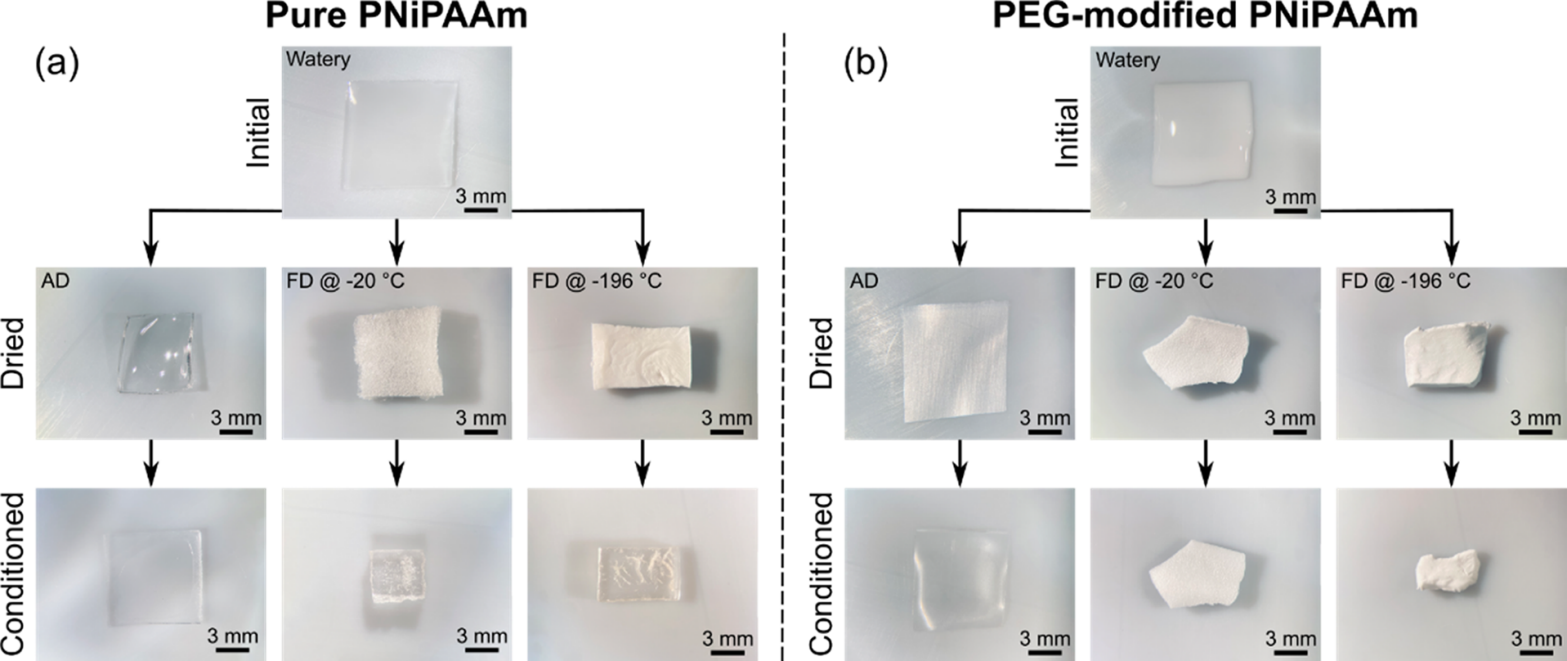
Optical microscopy images of (a) pure and (b) PEG-modified PNiPAAm in the initial state after drying (air-drying (AD) at room temperature or freeze-drying (FD) at −20 or −196 °C) and after conditioning in saturated water vapor for 24 h. The scale bar in all images is 3 mm. Please note that the depicted “initial” sample is the one which has undergone air-drying. The initial freeze-dried samples are different ones but not depicted here for clarity. They look similar to the initial example shown.

Figure 2 depicts optical microscopy images of pure and PEG-modified PNiPAAm hydrogels in the as-fabricated state, directly after drying under different conditions and after 24 h conditioning in saturated water vapor (*100% RH).

In the initial fully swollen state right after fabrication, the pure hydrogel is clear, while the PEG-modified one appears whitish opaque, indicating a more porous structure (top rows in Figure 2a,b). Two possible explanations have been proposed for this phenomenon. First, the long PEG molecules may introduce spatial obstruction during polymerization and cross-linking, resulting in a more porous structure within the PNiPAAm network after leaching. Second, the presence of PEG can induce phase separation among the PNiPAAm chains during polymerization, leading to the formation of macroporous and heterogeneous structures. Both factors contribute to the opaque appearance of the hydrogel material.^52^

For the pure sample, air-drying and subsequent conditioning lead to shrinking and reswelling with a transparent appearance in all steps. In contrast to that, the PEG-modified sample almost maintains its size when air-dried and shrinks only in the conditioning step. Furthermore, it then becomes transparent and looks very similar to the pure hydrogel.

For freeze-drying, a dependence of the resulting sample appearance on the freezing temperature and cooling rate is evident in pure PNiPAAm. While precise measurement of the cooling rate was not possible during the experiment, a comparative analysis of samples of similar size reveals that quenching in liquid nitrogen (−196 °C) results in a significantly accelerated cooling in contrast to −20 °C. When freezing occurs at a higher temperature of −20 °C, the sample appears rougher and more “porous” than its counterpart at −196 °C which exhibits a smoother surface.

In both cases, the samples are white and opaque. After conditioning, they both become transparent again, with the −20 °C sample exhibiting a larger shrinking than the one frozen at a lower temperature.

In the case of the PEG-modified PNiPAAm, the freeze-drying temperature does not lead to a noticeable difference in the appearance right after drying. However, the shrinking after conditioning is larger for the sample frozen at −196 °C than for the sample frozen at −20 °C. This is a reverse behavior compared to that of pure PNiPAAm. The appearance of the PEG-modified material remains white and opaque in all cases.

**Figure 3:**
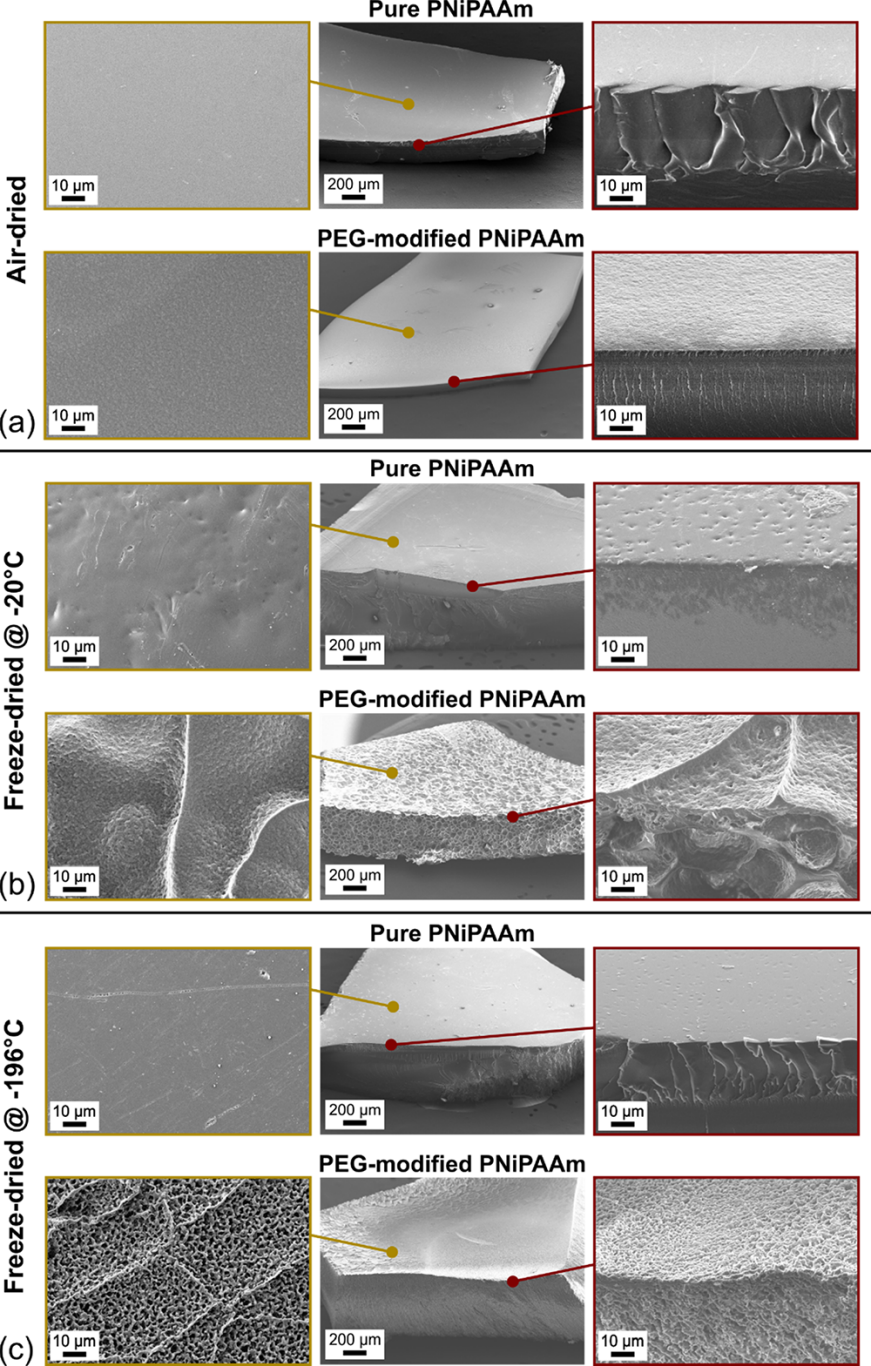
Scanning electron microscopy images of pure and PEG-modified PNiPAAm hydrogels after (a) air-drying and (b, c) freeze-drying at −20 and −196 °C, respectively. All samples have been conditioned in saturated water vapor for 3 days after drying to reach a stable equilibrium. The center images provide an overview, and left and right columns show a magnification of the surface and surface/cross-section transition as indicated.

These findings for sample appearance can be compared to the SEM images of the microstructures, which are depicted in Figure 3 as an overview as well as magnified images for surface and cross sections. All samples have been treated with the respective drying method and then conditioned in saturated water vapor for 3 days to reach an equilibrium state.

For air-drying, both samples (with and without PEG) exhibit a compact and dense inner structure. In all cases, the surface is nonporous but very smooth for the unmodified hydrogel, while it is rough and uneven for the modified counterpart.

In contrast, a clear difference between pure and PEG-modified PNiPAAm is visible for the freeze-dried samples. Regardless of the freezing temperature, the pure material exhibits an overall smooth surface without any pores but some dents and a densely packed internal structure. This is similar to the air-dried counterpart and is in accordance with the optical microscopy analysis.

The freeze-dried PEG-modified PNiPAAm shows a highly porous internal structure with a clear dependence of the pore diameter on the freezing temperature. For −20 °C, the pores are much larger than those for a freezing temperature of −196 °C.

An important difference can be observed for the surface: It appears to be porous for the sample frozen at −20 °C, but the magnified image reveals an uneven structure with features reminiscent of fault lines but only very few openings, which penetrate into the interior. Instead, the surface has formed a stable skin layer around the porous internal network (Figure 3b, lower right image). This is consistent with the reduced shrinking observed for this sample during conditioning, where the thicker walls between the pores and this skin layer result in a higher mechanical stability.

In contrast to that, freezing at −196 °C not only results in smaller pore sizes but also equal porosities of the surface and internal parts, forming a truly interconnected network. Due to this filigree structure, this sample shrinks more during conditioning (Figure 2). Please note that the finely porous structure can exhibit variations depending on the intrinsic porosity of the hydrogel, which is, for example, influenced by the amount of monomer and cross-linker.^47^

From these observations, the following conclusions can be drawn:

##### 3.1.1.1 Influence of PEG

The addition of PEG to the precursor solution results in hydrogel polymerization around these molecules. Once the PEG is washed out, the remaining voids increase the surface area of the hydrogel and make it more porous internally as well as on the surface, resulting in a whitish appearance. However, the obtained porous structure is stable only as long as the hydrogel is in a liquid environment. Once it is dried and conditioned, the polymer chains undergo relaxation and reconfiguration processes to reach an equilibrium state, which can result in a collapse of the porous structure,^48^ depending on the drying method as described in part (ii) (lower rows in Figure 3a–c).

FT-IR analysis was used to confirm the presence (after polymerization) and complete removal (after washing) of PEG to ensure that there were no residues that could influence the subsequent gravimetric experiments (Figure S1). For the unwashed PEG-modified sample, a sharp adsorption at 1100 cm^–1^ was observed, which corresponds to a −C–O–C– stretching vibration of PEG.^57^ This peak is not present in the pure PNiPAAm and the sample with 30-day washing, indicating an almost complete removal of the porogen. It should be noted that a thorough washing process, comprising at least 3 days of rinsing with a substantial amount of water, is essential to entirely eliminate the porogen.

Despite evidence of FT-IR results, it is possible that trace PEG residue might remain in the material as the long-chain polymer could potentially entangle with the PNiPAAm network during polymerization without actively participating in the reaction. In that case, residual PEG could have an influence on the freezing process. However, based on the FT-IR results, we assume that this is negligible in our case.

Furthermore, hydrogels with different PEG molecular weights of 600; 2,000; 10,000; and 35,000 g mol^–1^ were fabricated, and their microstructures were compared by SEM (see Figure S2). No notable difference was found, and since the purpose of the presented investigations was the porosity engineering of the hydrogel material, PEG with 10,000 g mol^–1^ was chosen for all further studies as an exemplary test candidate.

##### 3.1.1.2 Influence of Drying Method

*Air-drying* results in a nonporous structure after sample conditioning, independent of the addition of PEG. For the pure hydrogel, the expected shrinking and reswelling in dependence on the humidity condition is observed.

For the PEG-modified PNiPAAm, the imprinting with the PEG molecules during polymerization leads to a pore formation, which is stable during air-drying and prevents structural collapse due to the capillary forces when water molecules evaporate. Hence, no shrinking is observed after drying, but the polymer matrix is still stretched and cannot relax due to the high stiffness in this state. Consequently, the moisture during conditioning allows the polymer chains to reconfigure, closing the voids originally left by the PEG molecules and resulting in severe shrinking (Figure 2b). Hence, after conditioning, pure and modified PNiPAAm become similar in their appearance and microstructure (Figures 2 and 3a).

In *freeze-drying*, the sample is directly frozen in the swollen state, followed by sublimation of the ice at low pressure. In contrast to air-drying, there is no slow evaporation of water and, hence, no capillary force, which could collapse the internal structure.^50^ Instead, the water in the polymer matrix transforms into ice crystals, which can grow depending on the freezing temperature (see part (iii)). In any case, the freezing preserves the stretched polymer matrix from the liquid (i.e., swollen) state. As the material is fully dried afterward, the polymer chains cannot relax until the sample is conditioned in saturated water vapor. This enables movement and relaxation of the chains, resulting in shrinking in all cases, regardless of the addition of PEG.

For pure PNiPAAm, the transition from white (after freeze-drying) to transparent (after conditioning) clearly indicates that the voids created by the ice crystals during freezing have been closed by the reconfiguration of the polymer matrix. Therefore, no porous structure can be maintained (see the SEM image in Figure 3).

**Figure 4:**
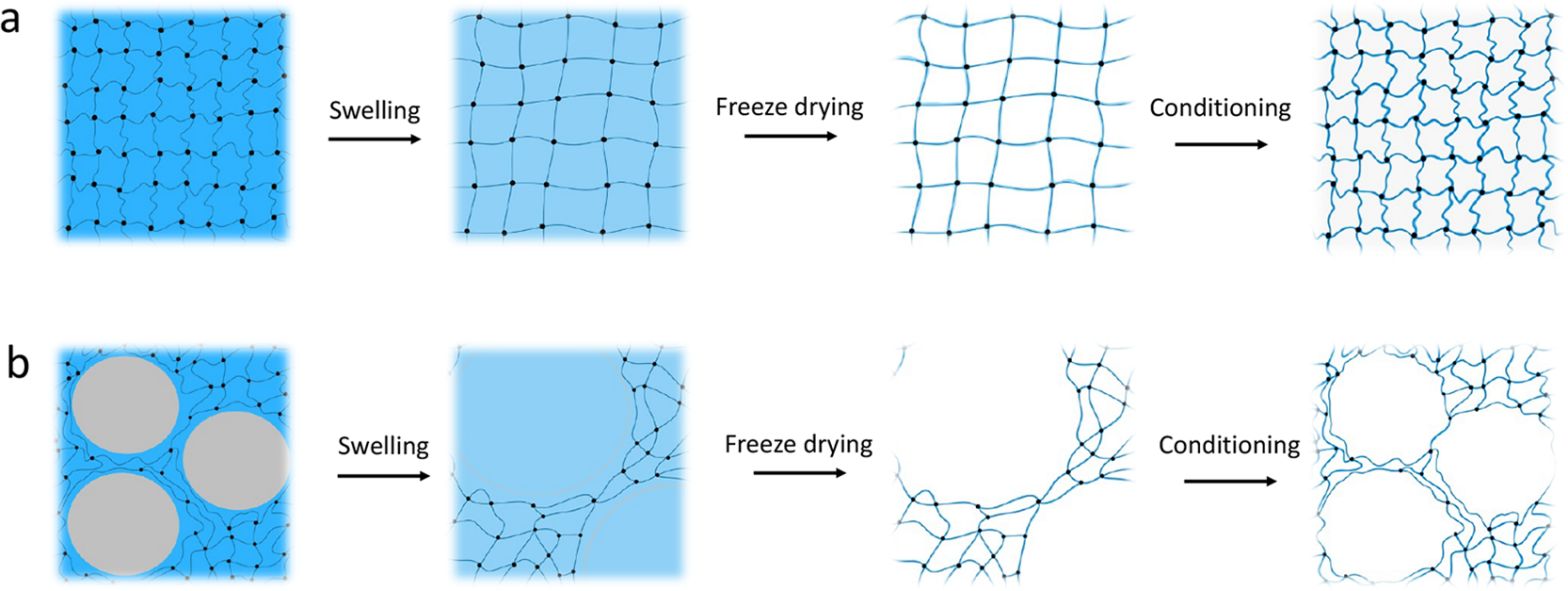
(a) Pure and (b) PEG-modified PNiPAAm hydrogel structures after swelling in liquid environment, freeze-drying, and conditioning. For easy understanding, long-chain PEG molecules are simplified and represented here as gray domains.

In the case of the PEG-modified PNiPAAm, the samples shrink in conditioning but the polymer matrix reconfigures to the imprinted structure, that is, the voids obtained from the PEG imprinting remain. This is confirmed by the white and opaque appearance as well as from the SEM images. The sketch in Figure 4 illustrates the described processes.

##### 3.1.1.3 Influence of Freeze-Drying Temperature

The freezing temperature modulates the formation of ice crystals. In the case of a very low temperature of −196 °C, the water inside the polymer matrix immediately turns into ice without time for crystal growth or much movement of the polymer chains. In principle, the intrinsic spaces in the polymer network are preserved and this happens in a similar way for the pure and modified hydrogel so that their appearance is similar immediately after drying (Figure 2). However, the fine-grained porous structure with homogeneous pore size and distribution is maintained only after conditioning for the PEG-modified material as indicated in the SEM images and final appearance (Figures 2 and 3).

At the higher freezing temperature of −20 °C, the ice crystals grow more slowly, and as the polymer chains remain mobile for some time, they are pushed into a stretched nonequilibrium state by the ice. After sublimation, the cavities formed by the ice remain as the polymer chains cannot relax due to the absence of water. Hence, the rough and whitish appearance of the pure PNiPAAm sample after freeze-drying.

In the case of the PEG-modified hydrogel, the voids left from the PEG imprinting already provide space for the ice to grow and the polymer chains are not being stretched much further. Therefore, not much difference is found in the macro-appearance of the two PEG-modified materials directly after freeze-drying at either higher or lower temperatures (Figure 2).

The impact of the temperature influence becomes evident only after conditioning: the sample frozen at −196 °C shrinks significantly more due to its finely porous inner and surface structure, while the material frozen at −20 °C is structurally more stable due to the surface skin layer and corresponding thicker polymer walls also in the internal part.

#### 3.1.2 Static Swelling Response from Gravimetric Measurements

**Figure 5:**
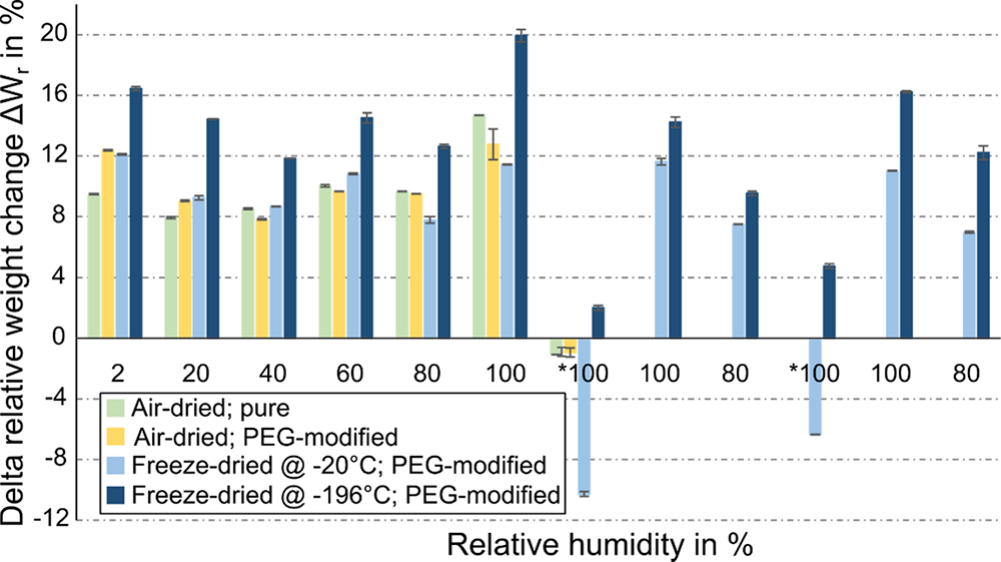
Delta relative weight change Δ*W*_r_ resulting from additional 100 ppm of acetone of pure and PEG-modified PNiPAAm hydrogels (dried by different methods) in varying relative humidity (calculated based on eq 2). Please note that *100 denotes saturated water vapor. All weights are mean values obtained from three consecutive measurements per sample, and standard deviations have been calculated accordingly.

In order to investigate how the different porous structures affect the performance of gas adsorption, gravimetric measurements in varying relative humidity without and with an additional 100 ppm acetone as the test analyte were conducted. As described in Section 2, samples were subjected to each condition for 24 h before being weighed and left to recover under cleanroom conditions for another 24 h. Figure 5 depicts the delta relative weight change Δ*W*_r_ for the different samples calculated by eq 2. Therefore, first, the change in sample weight solely due to relative humidity is obtained as a percentage value of the initial sample weight. Then, the measurement is repeated with the same humidity condition plus 100 ppm of acetone, resulting in a second weight change value (again, initial weight as reference). The difference between both weight changes is the resulting delta relative weight change Δ*W*_r_ and reflects the material’s response to acetone.

Four different types of samples were considered in these weighting measurements. Since the optical and microstructure analyses clearly indicate the independence of the drying method for the pure material, only air-dried unmodified PNiPAAm is considered. For the PEG-modified hydrogel, all three drying conditions are included in the comparison.

Furthermore, all samples were subjected to each individual humidity condition from 2 to *100% (saturated water vapor) and then the measurement was continued for two more cycles of reduced and increased RH for the two freeze-dried PEG-modified samples to study reversibility of the materials which showed the most significant changes.

The highest delta relative weight change with the presence of acetone was obtained with the material freeze-dried at −196 °C for all humidity conditions. Swelling was observed even in the case of saturated water vapor (i.e., *100% RH) where a strong competition for interaction sites at the polymer network between water and acetone gas molecules occurs.^44^ All other samples exhibited weight loss for this condition. What is most notable here is that the PEG-modified material frozen at −20 °C does not show an increased swelling response compared to its unmodified or air-dried counterparts despite having a porous internal structure. However, the SEM micrographs indicate skin formation on the surface with only very few pores compared with the −196 °C sample.

These observations clearly highlight the necessity of equal porosity of surface and bulk for gas sensing, which can only be obtained by the addition of PEG and freeze-drying at −196 °C.

Furthermore, the results from several cycles indicate a good reversibility and hence stability of the created porous structure as well as complete desorption of gas molecules (no accumulation). Please note that these gravimetric measurements take a long time (24 h for each condition with 24 h recovery in between), and therefore, fluctuations in clean room temperature and contaminants from the surrounding air may influence the sample, therefore impacting reproducibility during several measurement cycles. Hence, these experiments are only used to preliminarily assess the principle performance of the porosity-engineered materials as well as the stability of the porous structure. A more precise analysis of the sensing performance is only possible with advanced sensor platforms such as the piezoresistive pressure sensors described in Section 3.2.

From the bulk sample studies, it can be concluded that

(i) The combination of PEG-imprinting, freeze-drying, and conditioning is crucial for the creation and stabilization of a porous surface and interior structure of the hydrogel material in a gaseous environment. Either one alone is not sufficient and results in loss of porosity.
(ii) Freeze-drying at a lower temperature creates a more homogeneous porous structure (surface and inner part), which is beneficial for gas sensing applications.
(iii) In general, both freezing temperatures allow for the stabilization of the internal porous structure.

### 3.2 On-Chip Hydrogel Samples

#### 3.2.1 Continuous Swelling Response in Varying Acetone Concentrations

**Figure 6:**
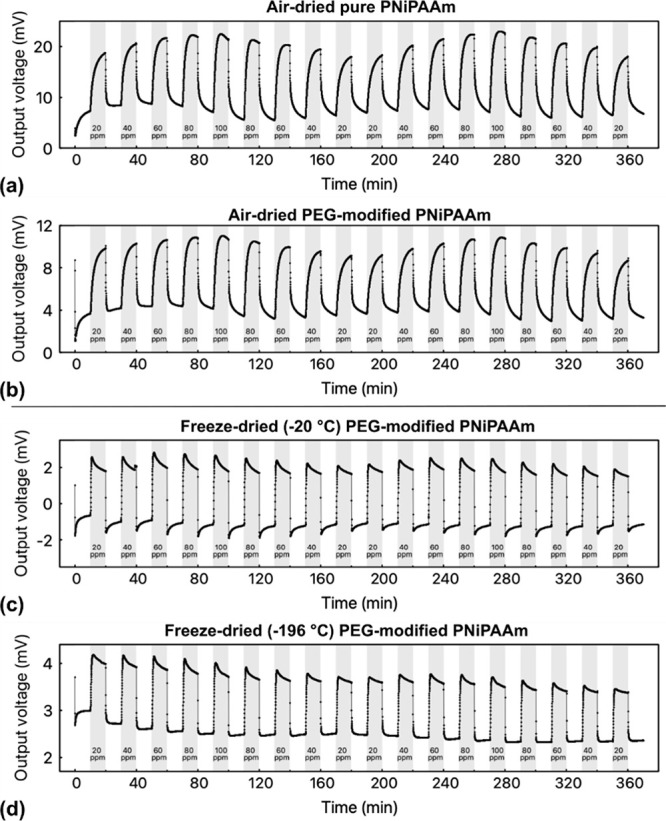
Pressure sensor chip output voltage for (a) air-dried pure PNiPAAm and (b–d) PEG-modified PNiPAAm with different drying conditions for cycling in varying acetone concentrations in a dried nitrogen atmosphere. After each concentration, the measurement chamber was purged by pure nitrogen flow for 10 min to allow the sample to recover and remove all residual acetone molecules.

Samples on piezoresistive pressure sensor chips were fabricated, as described in Section 2.2, and the same four types of materials as in the bulk studies were considered. The resulting continuous sensor output voltages are depicted in Figure 6 for several cycles of increasing and decreasing acetone concentrations between 20 and 100 ppm in a dried nitrogen atmosphere. Between each solvent concentration, the measurement chamber was purged with pure nitrogen flow for 10 min to remove all acetone molecules and allow the hydrogel to recover.

**Figure 7:**
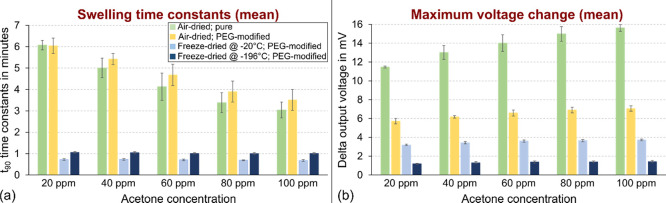
Pressure sensor chips response to varying acetone concentrations: (a) swelling time constants *t*_90_ and (b) maximum delta output voltages. The values have been calculated as means from several cycles, as depicted in Figure 6. The definition for time constant calculation and numerical values for both diagrams can be found in Figure S4, Tables S1 and S2.

The corresponding mean values for swelling time constants *t*_90_ and delta output voltages of the pressure sensor chips are depicted in Figure 7. The delta output voltages were calculated as the difference between the baseline and the value for each acetone concentration right before the nitrogen purge. Details of the time constant definition as well as the complete numerical data sets can be found in Figure S4 and Tables S1 and S2.

The ultimate goal of the presented research is to employ hydrogels as VOC sensing materials for human exhaled breath analysis, which typically contains high humidity levels exceeding 90% RH.^58^ However, for the pressure sensor experiments, a dried nitrogen atmosphere was chosen to avoid the influence of the cononsolvency effect, which has been reported to also occur in gaseous states.^59^ This effect leads to the PNiPAAm’s response being dependent on the ratio of water to acetone in the gas mixture.^60,61^ To simplify the initial investigation and focus on the effects of the porosity engineering strategies rather than on the interplay between water vapor and solvent molecules, we used dry nitrogen as the background instead of different humidity.

##### 3.2.1.1 Dynamic Behavior of Acetone Response

In all curves, a reproducible dependence of the output voltage on the acetone concentration can be observed. For the air-dried hydrogels (Figure 6a,b), the curve shapes are similar and exhibit a clear hysteresis: upon introduction of the acetone gas, the voltage increases strongly first and then creeps toward a steady state with a reduced slope. The creeping is more pronounced for lower acetone concentrations. This is the typical diffusion and viscoelastic creeping behavior known from hydrogel-based pressure sensors.^62−64^

For the freeze-dried samples (Figure 6c,d), the curve shapes look similar to one another but significantly differ from the air-dried ones. Almost no hysteresis is observed, and the sample responds equally fast to the introduction and removal of acetone gas. Furthermore, the creeping behavior is replaced by an “overshooting”, that is, the output voltage peaks at the introduction of acetone and then slightly decreases. This increased with increasing acetone concentration.

The underlying kinetics of this behavior require further investigations, but it can be assumed that the overshooting and subsequent relaxation are related to the porous structure: When exposed to the analyte gas, the molecules do not need time for diffusion processes but can immediately bind to the polymer, which they do due to the high affinity for the functional groups of PNiPAAm. After this immediate reaction, many more analyte molecules are present in the hydrogel network than in the surrounding atmosphere, leading to a concentration gradient and subsequent desorption of some of the molecules until an equilibrium is reached. Hence, the output voltage first peaks and then recedes to a steady-state value.

##### 3.2.1.2 Swelling Time Constants

The values for the swelling time constants (Figure 7a) support the above observations that the response of freeze-dried samples is much faster than that of air-dried ones.

Furthermore, a reduction of the response times with increasing acetone concentration is found, which is much more pronounced for air-dried samples. This can be explained by the denser inner structure (smaller surface area) and specifically the less porous surfaces for the air-dried materials, as discussed above. Therefore, for low acetone concentrations, it takes more time for the molecules to initiate a volume change, while the highly porous surface and internal structure of freeze-dried samples provide a large surface area. Consequently, higher acetone concentrations lead to the observed ∼50% reduction of the time constants in the case of air-dried materials, while the freeze-dried ones show only a slight decrease (see Table S1 for detailed numerical values).

**Figure 8:**
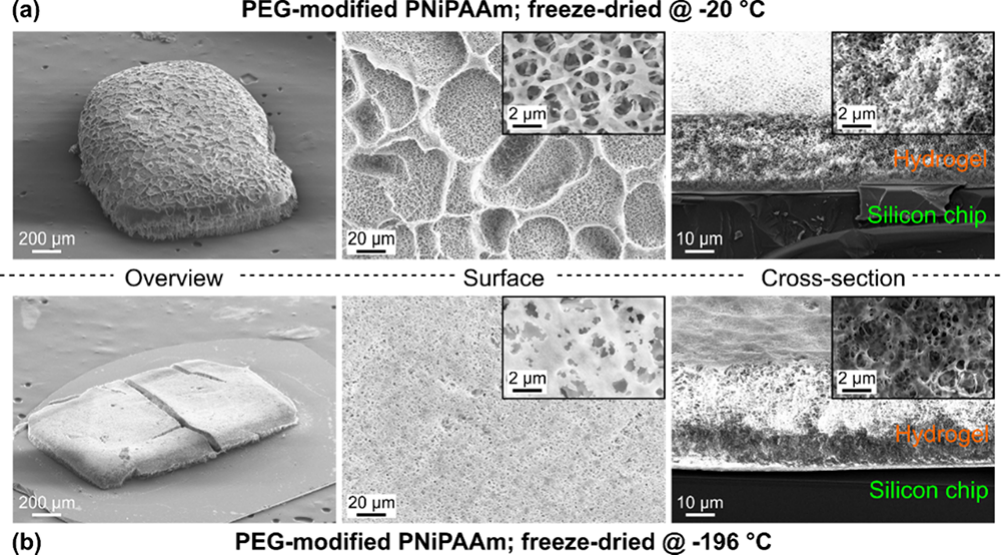
SEM images of PEG-modified freeze-dried hydrogels on chips frozen at (a) −20 and (b) −196 °C. The insets show magnification of the surface (middle) and inner structure (right).

This is furthermore supported by the SEM images depicted in Figure 8, where an equally porous surface and inner structure are visible for both freezing temperatures. This differs from what has been observed in the bulk samples where those frozen at −20 °C exhibited a skin formation and rather closed surface.

A likely explanation for this difference is that the on-chip hydrogels are very thin (less than 10 μm) compared with the 500 μm thick bulk samples. Hence, while the ice crystal growth in bulk materials mainly affects the interior porous network, in thin samples, they have a significant effect on the surface porosity, and skin formation is reduced or does not occur at all. The interior porous structure is similar for both freezing temperatures and is in accordance with the observations of the bulk samples.

##### 3.2.1.3 Magnitude of Output Voltage

By comparing the output voltage amplitudes depicted in Figure 7b, we can further confirm the impact of the different porosities. As evident from the microstructure analysis of the bulk samples, air-dried pure PNiPAAm is not porous and, hence, has a large contact area with the silicon membrane of the pressure sensor. Consequently, the swelling-induced force transmission is very high, resulting in a large deformation of the membrane and corresponding high output voltage.

All PEG-modified samples exhibit a significantly reduced voltage change, even the air-dried one, which does not have a porous structure. This can be explained as follows: When the precursor solution is polymerized on the chip, the PEG molecules will also occupy space at the interface between the hydrogel and silicon membrane. Once the PEG is removed after washing and the samples undergo the drying and conditioning steps, the polymer chains reorganize as described above. However, the adhesion at the interface to the silicon somewhat restricts chain movement; hence, the voids left by the PEG cannot fully close, even under air drying. This results in a reduced overall contact area between the polymer material and pressure sensor membrane and therefore reduced deformation force and output voltage. For the freeze-dried samples, this reduction in force is even greater due to the overall more porous structure, resulting in the observed low output voltages. In this regard, the fine pores obtained by freezing at −196 °C can be detrimental to the signal strength of the pressure sensor read-out method. Furthermore, rheological measurements of pure and PEG-modified PNiPAAm indicate a reduced modulus in the case of the PEG modification (see Figure S5), which likely also contributes to the reduced forced transmission on the pressure sensor membrane.

Therefore, it needs to be noted that the output voltage magnitude mainly reflects the properties of the transducer–hydrogel interface and not the hydrogel swelling behavior itself. For future sensor applications, a different transduction method for the hydrogel’s swelling state may be more beneficial.

Overall, the on-chip measurements clearly confirm the findings from the bulk studies: the combination of PEG modification and freeze-drying is necessary to create a stable porous structure, which enhances the gas sensing performance and response of the hydrogel material. PEG modification alone is not sufficient, as the similarity of the curves in Figure 6a,b indicates.

#### 3.2.2 Hydrogel Integrity on Chip

**Figure 9:**
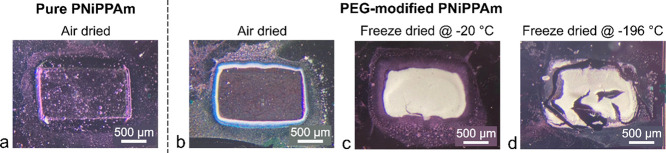
Optical microscopy images of (a) air-dried pure PNiPAAm and (b–d) PEG-modified PNiPAAm with different drying conditions after several swelling cycles in acetone gas.

The drying method and specifically the freezing temperature have a clear effect on the sample appearance. Figure 9 depicts optical microscopy images of the four different chip samples after several cycles in an acetone atmpsphere. While both air-dried hydrogels look similar (transparent as expected for nonporous material), a significant difference is found for the two freeze-dried ones. The material frozen at −20 °C is intact with only some slight cracks, while the one freeze-dried at −196 °C is fractured. These different morphologies can also be seen in the overview SEM images in Figure 8. This is attributed to the distinct temperature coefficients of silicon and hydrogel, which results in mechanical stress at the interface during freezing and subsequent crack formation. For the higher freezing temperature of −20 °C, the stress is reduced due to the slower freezing, which allows the polymer chains to rearrange, as long as there is still enough liquid to enable movement.

When the hydrogel is reversibly swelling and shrinking, the cracks can become larger and lead to a significant fracture of the material (Figure 9d). Hence, in terms of future sensor development, the choice of a suitable transduction principle for the hydrogel’s swelling state needs to consider this aspect of different thermal expansion coefficients.

Furthermore, the freeze-dried samples tend to fall off the chip after drying. This is attributed to the highly porous structure and reduced interface forces, in combination with the mechanical stress induced during freezing. Therefore, a drop-coating step has been integrated into the fabrication process for freeze-dried on-chip samples, as depicted in Figure 1. Thereby, a drop of deionized water is applied to the hydrogel and left there for 1 min before being removed with a clean room tissue. Immediately afterward, the sample is moved on to the freezing step.

The underlying mechanism of why drop-coating prevents hydrogel detachment from the silicon membrane after drying and conditioning needs to be investigated further. At this point, we note that this process leads to almost 100% yield of intact samples, even if fractured, as in the case of −196 °C freezing temperature.

Additionally, we have studied the long-term adhesion properties by comparing sensor performance right after sample fabrication and 1 year later. No degradation or delamination of the hydrogel has been found, and the sensor output signals are very similar. The corresponding chip measurements can be found in Figure S6.

From all of these observations, the following conclusions can be drawn for the on-chip samples:

(i) The combination of PEG-modification and freeze-drying is crucial for enhancing gas sensing performance in terms of response time.
(ii) For thin samples (thickness of less than 10 μm), no skin formation occurs and freezing at −20 °C results in an equally porous surface and internal structure, same as −196 °C.
(iii) A freezing temperature of −20 °C is more suitable for thin samples attached to a surface as it preserves structure integrity (almost no cracks).
(iv) Drop coating with DI water before freezing significantly improves adhesion between hydrogel and silicon surface.

## 4 Conclusions

Recently, poly(*N*-isopropylacrylamide) (PNiPAAm) was identified as a promising candidate for smart hydrogel-based gas sensing in nonliquid environments. In the study presented here, the focus is on tailoring the material’s porosity to enhance the gas sensing capabilities and performance by increasing the surface area and specifically adjusting the surface and internal porosity.

In contrast to a liquid environment, which provides fairly stable surrounding conditions, a gaseous atmosphere can feature large variations in humidity, ranging from almost completely dry to saturated water vapor. This poses a challenge for the preservation of structural integrity of the material since the mobility of the polymer chains is strongly affected by the surrounding conditions.

In our study, we have investigated the feasibility of polyethylene glycol (PEG) as a porogen as well as different drying procedures to achieve (i) an equally porous surface and internal structure, (ii) stabilization of the created pores for gaseous conditions, and (iii) enhanced responsiveness in terms of swelling time constants and strength of volume change in PNiPAAm hydrogel.

Therefore, bulk and thin film samples on a piezoresistive pressure sensor chip have been fabricated with and without PEG, followed by air-drying or freeze-drying at either −20 or −196 °C. All samples were characterized with regard to their appearance and microstructure by optical and scanning electron microscopy during the various fabrication stages. Furthermore, the response to the test analyte gas acetone was studied by gravimetry for the bulk samples and through continuous electrical measurements of the pressure sensor chip’s output voltage for the thin film samples.

These studies resulted in the following conclusions with regard to the aforementioned aims:

1. The combination of PEG, freeze-drying, and conditioning in saturated water vapor is crucial to achieve a stable porous structure of the hydrogel, which is maintained in all gaseous conditions.
2. The porosity engineering enables an enhanced gas sensing performance of PNiPAAm hydrogel and offers great potential for extension of smart hydrogel applications to gaseous environments.
3. For bulk samples of several 100 μm thickness, the freezing temperature affects the internal pore size (large for −20 °C, small for −196 °C) but a porous surface connected to the internal part can only be achieved by freezing at −196 °C.
4. For thin film samples (thickness <10 μm), both freezing temperatures result in similar a porous surface and internal structure.
5. When the hydrogel is fabricated on a substrate, the porous structure can be detrimental to adhesion, and furthermore, largely different temperature coefficients of hydrogel and substrate material can lead to fracturing of the hydrogel. Hydrogel surface adhesion on silicon can be substantially improved by drop-coating the polymer with DI water for several minutes before the freeze-drying procedure.

The present study was carried out for a PNiPAAm hydrogel and the test analyte gas acetone. However, the developed fabrication procedures and respective insights are more general in nature and can be applied for porosity engineering of any other hydrogel material to improve gas sensing performance and stability specifically in a gaseous environment.

Furthermore, in comparison to other materials commonly used for VOC sensing, the hydrogel synthesis in this study is remarkably straightforward and uncomplicated. The polymer properties can be easily tailored and customized to specific requirements by adding other active particles. Unlike inert polymer matrices often used in gas sensing, smart hydrogels such as PNiPAAm exhibit a high affinity for various organic gases. This unique characteristic may enable a combined sensing function of both matrix and filler particles, potentially leading to enhanced synergetic effects. This will be explored in our future research, where we will focus on studying the capabilities of porous PNiPAAm to discriminate between different test analyte gases and selectivity and specificity with regard to gas mixtures, as well as other sensing applications.

## Supporting Information Available

Detailed description of gravimetric measurements of bulk samples; FT-IR spectra for determination of PEG presence; SEM images of PEG-modified freeze-dried (−196 °C) PNiPAAm hydrogels with different PEG molecular weight; fabrication process of hydrogels on chip; definitions of time constants for chip-measurements; mean values of swelling time constants for on-chip samples; mean values of delta output voltages for on-chip samples; rheological test results of pure and PEG-modified PNiPAAm samples; and long-term stability of on-chip samples (PDF)
