# Supplementary material for: Porosity Engineering of Dried Smart Poly(N-isopropylacrylamide) Hydrogels for Gas Sensing
Source: Biomacromolecules. 2023 Dec 4;25(5):2715–27. doi: 10.1021/acs.biomac.3c00738 (PMC11094736; doi:10.1021/acs.biomac.3c00738)
Supplement: Supplementary file 1 — bm3c00738_si_001.pdf [file bm3c00738_si_001.pdf]

Supporting Information

Porosity Engineering of Dried Smart Poly(*N*-isopropylacrylamide) Hydrogels for Gas Sensing

Sitao Wang[1], Chen Jiao[2], Gerald Gerlach[1], Julia Körner[3]*

1 Institute of Solid-State Electronics, Dresden University of Technology, 01062 Dresden, Germany

2 Leibniz-Institut für Polymerforschung Dresden e.V., Hohe Straße 6, 01069 Dresden, Germany

3 Institute of Electrical Engineering and Measurement Technology, Leibniz Universität Hannover, 30167 Hannover, Germany

*koerner@geml.uni-hannover.de

Detailed description of gravimetric measurements of bulk samples

After conditioning, the bulk samples were broken into small pieces (thickness 500 µm; irregular shape as precise cutting is not possible; (5–10) mg per piece) and placed into a 2 mL Eppendorf tube which was put into a sealed measurement chamber with the predefined gaseous conditions. The sample was exposed to a condition for 24 h with the tube lid open so that the hydrogel could reach an equilibrium. Afterwards, the tube was taken out, the lid immediately closed to preserve the sample state and environment and tube plus sample were weighed by a Mettler Toledo XP26 analytical balance (Switzerland; sensitivity of 1 µg). After completion of the weighing measurement, the tube lid was reopened and the sample left to recover under ambient cleanroom conditions for another 24 h before the procedure was repeated with the next gaseous condition.

Gaseous environments with different relative humidity (RH) ranging from zero percent to saturated (RH = [0; 20; 40; 60; 80; 100; *100] %) were created in the chamber by employing humid vapor flow through a bubbler and a commercial humidity sensor (B + B sensors, Hytelog-RS232, Germany). It needs to be noted that we distinguish between 100% RH created by the humid gas flow and a saturated 100% condition created by a water reservoir in the chamber. The latter is denoted as *100% RH for the remainder of the text and refers to an excess abundance of free water vapor molecules. In contrast to that, the amount of water molecules is still limited for 100% RH.

In order to add an organic test gas to the chamber with a predefined humidity, absolute acetone was injected. The concentration of organic gas in the chamber can be calculated according to [Guo2018, Wang2023]:

$$\frac{c}{\mathbf{ppm}} = \frac{\frac{24.22}{\mathbf{L/mol}} \cdot \frac{T}{\mathbf{K}} \cdot \frac{\rho_\mathrm{f}}{\mathbf{g/mL}} \cdot \frac{V_\mathrm{f}}{\mathbf{mL}}}{\frac{273}{\mathbf{K}} \cdot \frac{M}{\mathbf{g/mol}} \cdot \frac{V}{\mathbf{L}}} \cdot 10^3 \qquad [S.1]$$

[Guo2018] Guo, H.; Gao, Y.; Liu, T. A Theoretical Study of the VOC Sensor Based on Polymer-Coated Diaphragm Embedded with FBAR. *Measurement* **2018**, 129, 206–210.

[Wang2023] Wang, S.; Gerlach, G.; Körner, J. A Study of Smart Hydrogels as Sensing Elements in Gaseous Environment for VOC Detection. *Polymer* **2023**, 278, 126009.

FT-IR spectroscopy of bulk samples

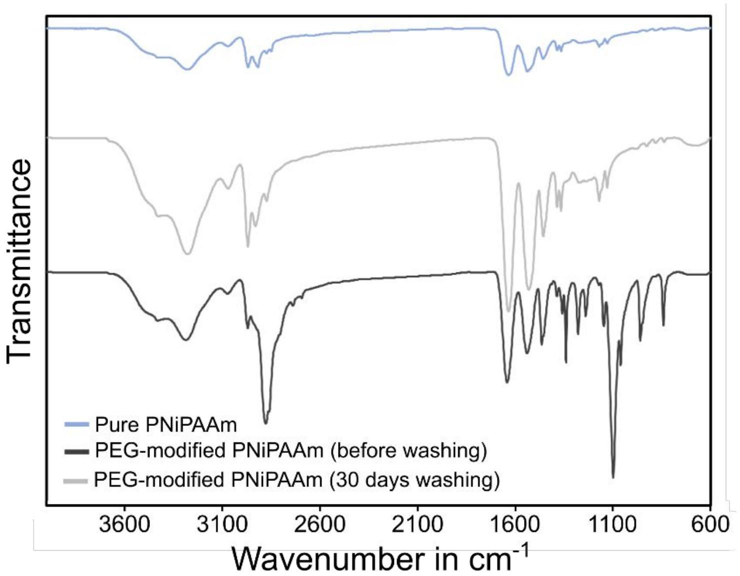

Figure S1. FT-IR spectra of pure PNiPAAm and its PEG-modified counterpart without and with different washing times in deionized water.

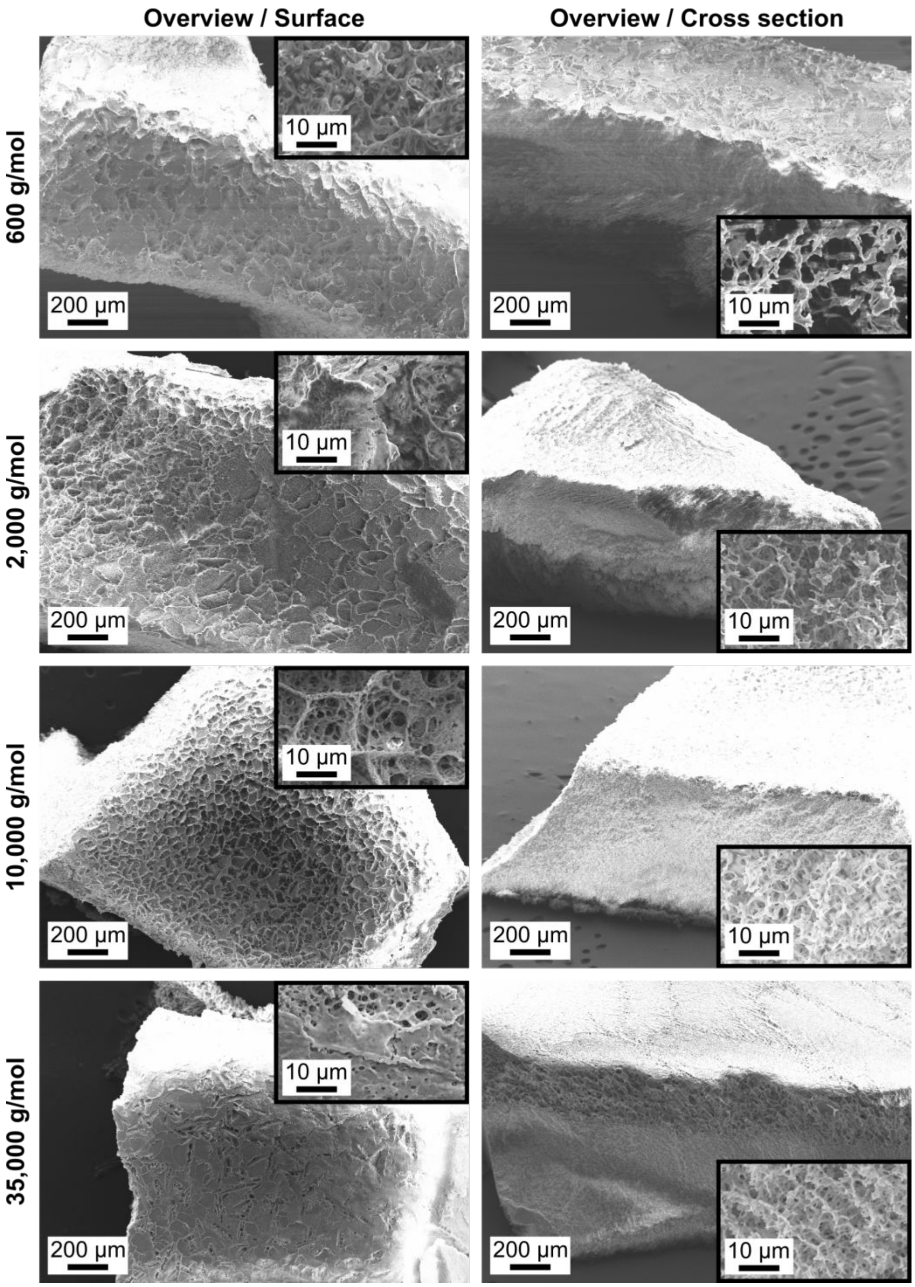

Figure S2. SEM images of PEG-modified PNiPAAm hydrogel freeze-dried at −196 ˚C after conditioning, with different PEG molecular weights of (600; 2,000; 10,000; 35,000) g/mol. For each sample the surface (left column) and cross section (right column) are shown with the insets providing a magnified view of the respective area.

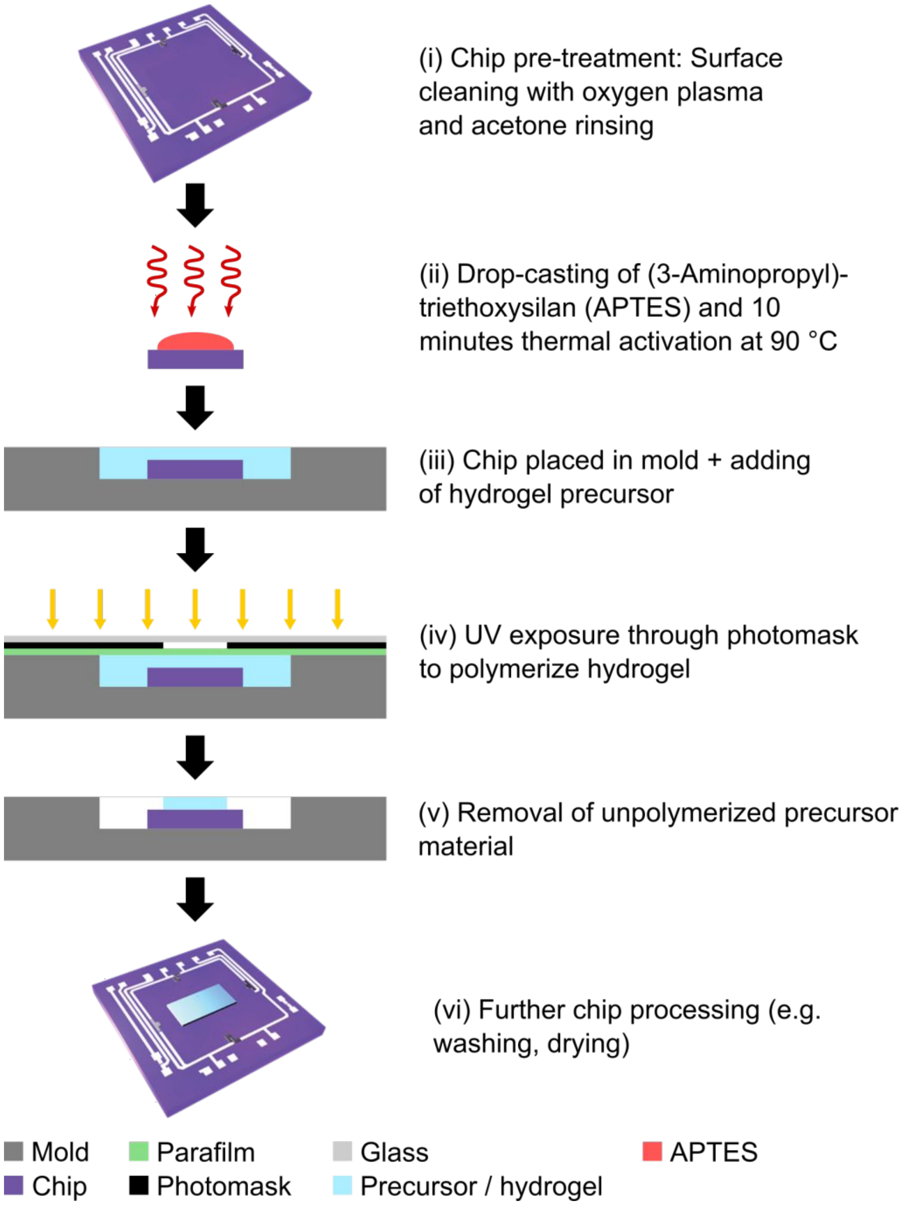

Figure S3. Process flow for on-chip hydrogel fabrication.

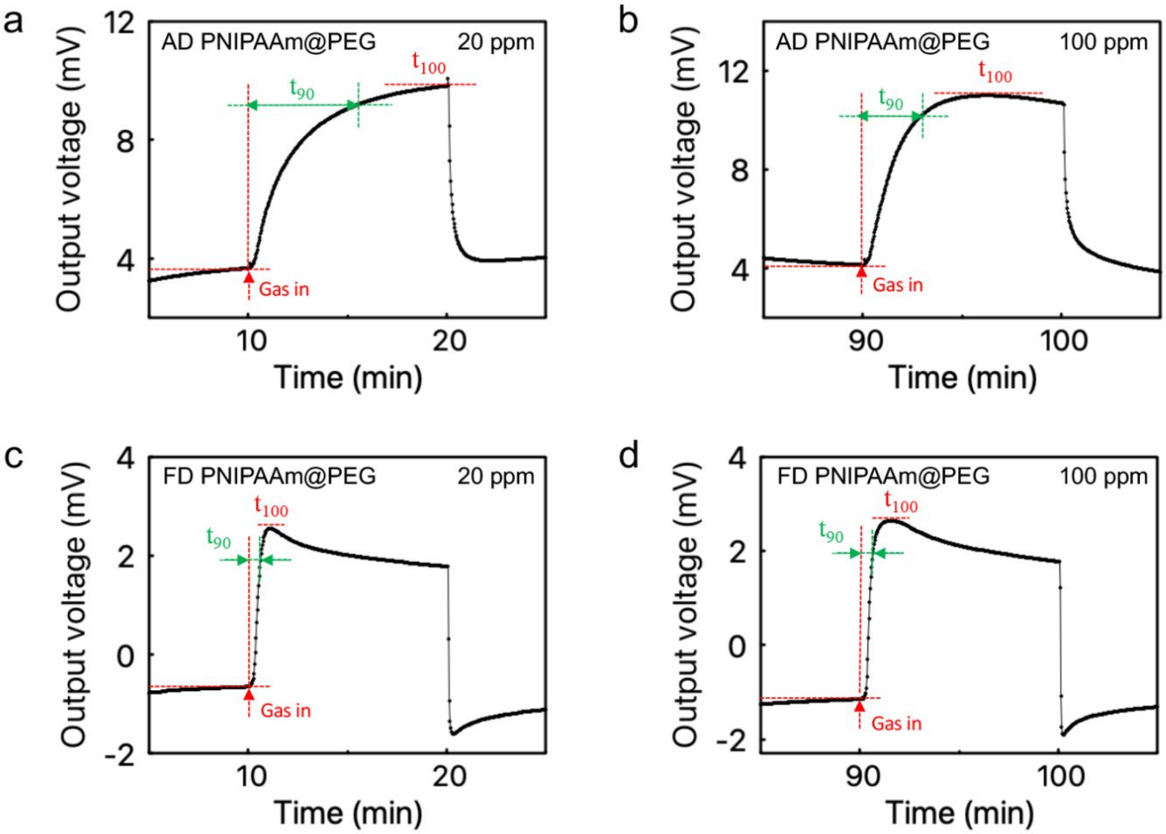

Figure S4. Relevant parameters and definition for analysis of the t_{90} response time for on-chip hydrogel samples. Exemplarily depicted are air-dried (AD) and freeze-dried (FD, –20 ˚C) PEG-modified PNiPAAm in response to (a, c) 20 ppm acetone and (b, d) 100 ppm acetone. The time interval between two consecutive data points is 2 s.

Table S1. Response time t_{90} of pressure sensor chips with different hydrogel samples to varying acetone concentrations. These are mean values obtained from several measurement cycles as depicted in figure 6 in the main text. The definition of the t_{90} time constant can be found in figure S4.

	**Chip response time (t_{90}) in minutes**				
Mat. | **Ace. conc.**	**20 ppm**	**40 ppm**	**60 ppm**	**80 ppm**	**100 ppm**
Pure, air-dried	6.07 ± 0.22	5.01 ± 0.46	4.13 ± 0.64	3.38 ± 0.47	3.04 ± 0.37
PEG-modified, air-dried	6.04 ± 0.36	5.42 ± 0.26	4.68 ± 0.50	3.90 ± 0.49	3.51 ± 0.49
PEG-modified, freeze-dried @ –20 ºC	0.72 ± 0.04	0.73 ± 0.04	0.71 ± 0.04	0.69 ± 0.02	0.68 ± 0.04

PEG-modified, freeze-dried @ –196 ºC	1.06 ± 0.04	1.04 ± 0.05	1.01 ± 0.03	1.00 ± 0.05	1.01 ± 0.04

Table S2. Delta voltages Δ *V* of pressure sensor chips with different hydrogel samples for varying acetone concentrations. These are mean values obtained from several measurement cycles as depicted in figure 6 in the main text.

	**Chip delta output voltage in mV**				
Mat. | **Ace. conc.**	**20 ppm**	**40 ppm**	**60 ppm**	**80 ppm**	**100 ppm**
Pure, air-dried	11,47 ± 0.11	13,02 ± 0.73	14,02 ± 0.89	14,99 ± 0.78	15,62 ± 0.36
PEG-modified, air-dried	5,72 ± 0.29	6,18 ± 0.14	6,58 ± 0.31	6,89 ± 0.30	7,06 ± 0.28
PEG-modified, freeze-dried @ –20 ºC	3,20 ± 0.07	3,43 ± 0.12	3,60 ± 0.12	3,65 ± 0.12	3,72 ± 0.09
PEG-modified, freeze-dried @ –196 ºC	1,18 ± 0.06	1,30 ± 0.12	1,37 ± 0.11	1,40 ± 0.10	1,42 ± 0.12

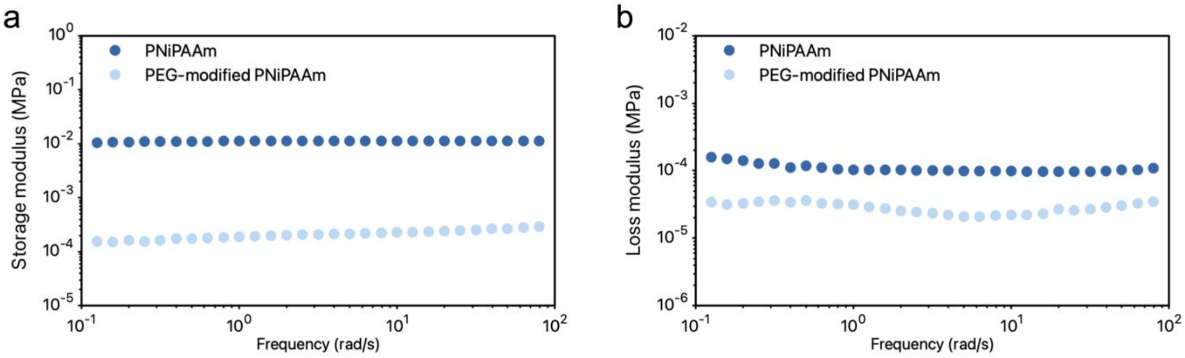

Figure S5. (a) Storage and (b) loss moduli for pure and PEG-modified PNiPAAm hydrogels. Measurements were conducted with a TA Instruments ARES-G2 rheometer in a configuration of two parallel stainless-steel plates with a diameter of 25 mm and a fixed oscillatory strain of 1% in a dynamic frequency sweep from 0.1 to 100 rad/s at 22 °C.

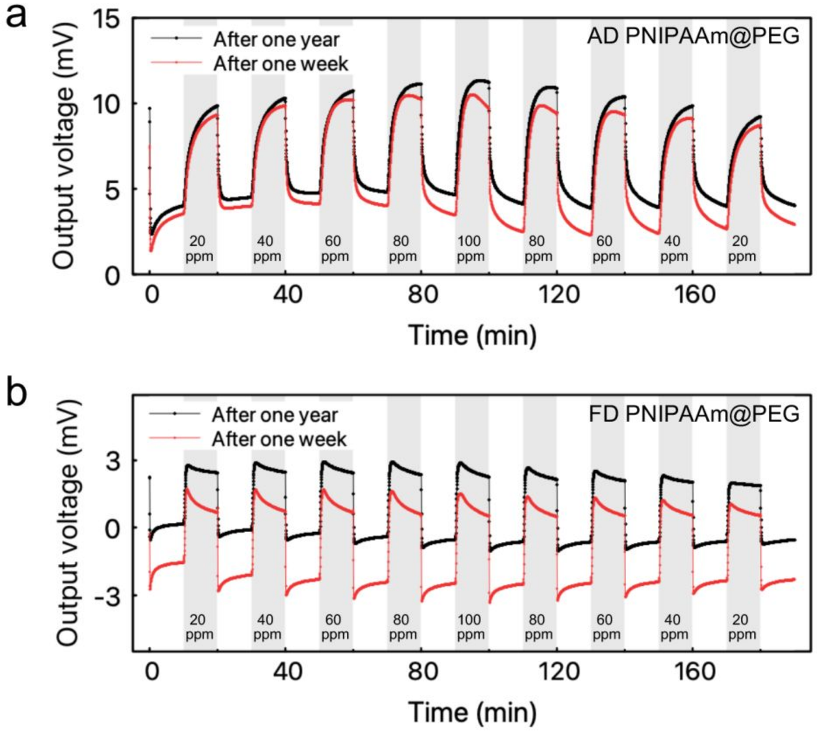

Figure S6. Measured output voltage of the piezoresistive pressure sensor chips of (a) air-dried (AD) and (b) freeze-dried (FD, –20 ˚C) PEG-modified PNiPAAm hydrogel one week and one year after fabrication. Samples were stored in a container under cleanroom conditions (22 ˚C; 45% relative humidity) in a dry state.

The behavior and curve shapes are reproducible and similar, indicating a good stability of the material itself and also adhesion to the pressure sensor membrane. The constant offset between the two curves in (b) is due to the electrical connection of the sensor to the measurement setup.
